# Lacosamide Use After Traumatic Brain Injury: A Scoping Review

**DOI:** 10.7759/cureus.112117

**Published:** 2026-07-06

**Authors:** Hiroaki Taniguchi, Seigo Yamada

**Affiliations:** 1 Department of Internal Medicine, Nishiguchicho Shin-ai Clinic, Toyohashi, JPN; 2 Medical Unit, Amphibious Rapid Deployment Brigade, Sasebo, JPN

**Keywords:** acute symptomatic seizures, lacosamide, neuroprotection, post-traumatic seizures, scoping review, seizure prophylaxis, traumatic brain injury

## Abstract

Lacosamide (LCM) is a newer antiepileptic drug that enhances slow inactivation of voltage-gated sodium channels and modulates collapsin response mediator protein-2, suggesting potential roles in seizure control and axonal protection after traumatic brain injury (TBI). However, its role in TBI remains unclear. This scoping review aimed to characterize preclinical and clinical research on LCM use in TBI, focusing on seizure prophylaxis, seizure treatment, and potential neuroprotective effects.

A comprehensive search of MEDLINE, Cochrane Central Register of Controlled Trials (CENTRAL), Cumulative Index of Nursing and Allied Health (CINAHL), Web of Science, and ClinicalTrials.gov was conducted. Studies involving human patients or animal TBI models evaluating acute LCM use were included, and data were charted descriptively.

Ten studies were included, comprising five preclinical and five clinical investigations. Preclinical studies evaluated seizure-related, electrophysiological, histological, inflammatory, and behavioral outcomes in experimental TBI models. Clinical studies included two randomized trials, two observational cohorts, and one case report involving patients with moderate to severe TBI. LCM was administered for seizure prophylaxis or treatment. Seizure-related and safety outcomes were reported across clinical studies, whereas neurological and functional outcomes were less consistently assessed.

Overall, evidence supporting LCM use in acute TBI remains limited, with differences between preclinical and clinical outcome domains.

## Introduction and background

Traumatic brain injury (TBI) is a leading cause of acute neurological morbidity and mortality worldwide. Acute symptomatic seizures and the development of post-traumatic epilepsy are frequent neurological sequelae of TBI, particularly in patients with moderate to severe injury or intracranial hemorrhage [1,2]. These seizures not only disrupt cerebral homeostasis but may also contribute to secondary brain injury and worsen long-term neurological outcomes [3]. Although current guidelines recommend short-term seizure prophylaxis in high-risk TBI patients, the optimal choice of antiepileptic drugs (AEDs) remains debated, particularly with respect to drug tolerability, pharmacokinetics, and potential neuroprotective effects [4-6].

Lacosamide (LCM) is a third-generation AED with a unique mechanism of action that enhances the slow inactivation of voltage-gated sodium channels and modulates collapsin response mediator protein-2 (CRMP-2), a molecule implicated in axonal guidance and neuroplasticity [7,8]. This dual mechanism has generated interest in the potential neuroprotective role of LCM following TBI. Preclinical studies using TBI models suggest that LCM may attenuate neuroinflammation and neuronal damage, with some studies reporting improvements in motor and cognitive outcomes [9,10]. However, these findings are variable and appear dependent on dosing and timing, leaving the translational relevance of these observations uncertain.

In clinical practice, LCM has been used off-label for seizure prophylaxis or treatment following TBI, particularly in cases where conventional agents such as phenytoin or levetiracetam are contraindicated or poorly tolerated [11]. However, the available human evidence in this context remains limited and consists predominantly of retrospective cohort studies. Although several systematic reviews and meta-analyses have examined the use of LCM in acute seizures and status epilepticus, few have focused specifically on patients with TBI [12]. A phase IV randomized controlled trial comparing LCM with levetiracetam for early post-traumatic seizure prevention is currently underway, underscoring the ongoing clinical equipoise in this area [13].

Despite the accumulation of both preclinical and clinical studies, the literature on LCM use in TBI has not been systematically mapped with respect to study designs, populations, interventions, and reported outcomes. Consequently, the structure and extent of the existing evidence base, as well as remaining knowledge gaps, remain unclear. The objective of this scoping review is to map the preclinical and clinical literature on LCM use in patients with TBI, with a focus on study characteristics, populations, interventions, and reported outcomes.

## Review

Methods

Conduct and Reporting of the Review

The conduct and reporting of this scoping review followed the Preferred Reporting Items for Systematic reviews and Meta-Analyses extension for Scoping Reviews (PRISMA-ScR) guidelines and the Joanna Briggs Institute (JBI) guidance for scoping reviews [14,15]. The protocol for this review was prospectively registered on the Open Science Framework to enhance transparency and minimize duplication of research.

Identifying the Research Question

The aim of this scoping review was to map the extent and nature of the available preclinical and clinical evidence regarding the use of LCM in the context of TBI. The review was guided by the following question:

What is the extent and nature of the evidence regarding the use of LCM in patients with TBI for seizure prophylaxis, seizure treatment, or neuroprotective purposes?

Search Strategy

The literature search was structured using the Population-Concept-Context (PCC) framework recommended for scoping reviews. An initial limited search of MEDLINE (via PubMed) was conducted to identify relevant articles and to refine the search strategy through analysis of keywords and index terms. The finalized search strategy was then adapted and applied to the following electronic databases: MEDLINE (via PubMed), the Cochrane Central Register of Controlled Trials (CENTRAL), Cumulative Index of Nursing and Allied Health (CINAHL) (via EBSCOhost), and Web of Science (Core Collection). Gray literature sources included ClinicalTrials.gov. Reference lists of all included studies were manually screened to identify additional eligible records. Searches were conducted from database inception to the date of the final search. Only studies published in English were included. Full search strategies for all databases are provided in Supplementary Appendix S1.

Eligibility Criteria

Eligibility criteria were defined a priori in accordance with the PCC framework.

Population

Studies involving human participants of any age with a diagnosis of TBI (mild, moderate, or severe) were eligible. Given the variability in TBI definitions across the literature, no restrictions were imposed on specific diagnostic criteria, severity scales, or neuroimaging findings. Studies including patients with acute symptomatic seizures or post-traumatic epilepsy were eligible. In addition, animal models of TBI were included to capture preclinical evidence relevant to mechanistic and pathophysiological investigation. Studies exclusively examining chronic epilepsy without a traumatic etiology were excluded.

Concept

Eligible studies examined the use of LCM for one or more of the following purposes: (a) seizure prophylaxis, (b) seizure management, including status epilepticus, or (c) investigation of neuroprotective or pathophysiological effects, provided that TBI was the primary clinical or experimental condition under investigation. Studies in which LCM was used for epilepsy unrelated to trauma were excluded.

Context

All clinical and experimental settings were considered, including emergency departments, intensive care units, neurocritical care units, rehabilitation settings, and laboratory-based experimental environments. No restrictions were applied with respect to geographic region, healthcare system, or study setting.

Types of Sources

Eligible sources included experimental and quasi-experimental studies, observational studies, descriptive studies (case reports and case series), and basic science studies involving animal models or cellular mechanisms. Systematic reviews, narrative reviews, and text or opinion papers were included for contextual mapping but were not considered primary evidence sources.

Selection Process

All identified records were imported into EndNote 20 (Clarivate Analytics, PA, USA) for duplicate removal and subsequently uploaded to Rayyan (Rayyan Systems Inc.) for screening. Two reviewers independently screened titles and abstracts in a blinded manner. Full texts of potentially eligible studies were retrieved and assessed against the predefined eligibility criteria. Discrepancies were resolved through discussion between the two reviewers. The study selection process was documented using a PRISMA-ScR flow diagram.

Data Extraction, Synthesis, and Presentation

Data were extracted using a standardized data charting form developed in Microsoft Excel, as described in Supplementary Appendix S2. Extracted variables included publication characteristics, study design, population or experimental model, TBI definition and severity, details of LCM exposure (indication, dose, timing, and route), comparator treatments where applicable, and reported outcomes. Outcomes were recorded as reported by the original authors and included seizure-related outcomes, neurological or functional outcomes, mortality, and safety outcomes. The data charting form was piloted on a subset of studies prior to full extraction. Data were synthesized descriptively and presented in tabular and narrative formats, with studies grouped by preclinical versus clinical design and by indication for LCM use. No quantitative synthesis or meta-analysis was performed.

Critical Appraisal

In accordance with JBI guidance for scoping reviews, no formal risk-of-bias assessment was conducted [14].

Results

Selection of Sources of Evidence

**Figure 1:**
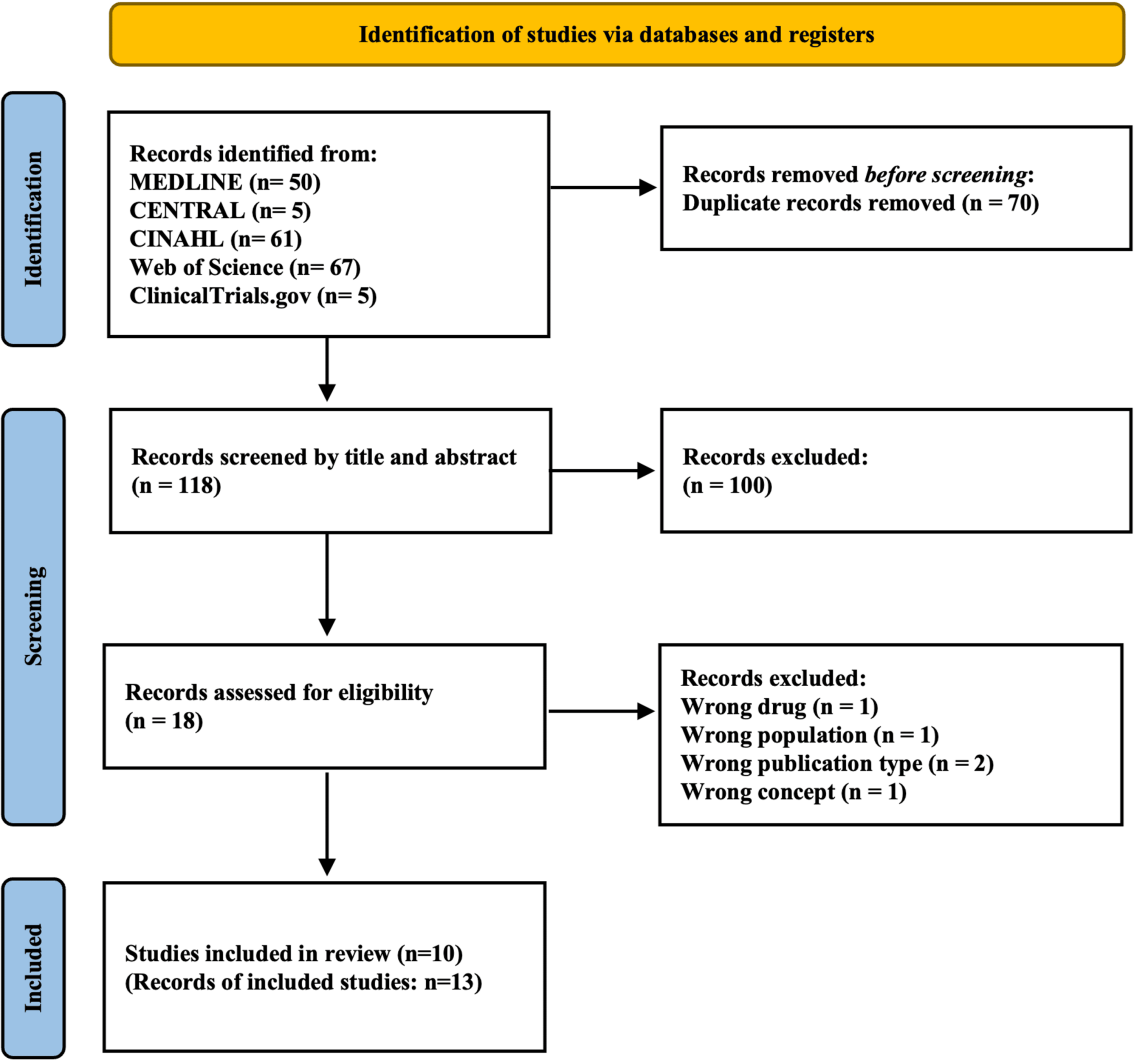
PRISMA-ScR flow diagram. PRISMA-ScR: Preferred Reporting Items for Systematic reviews and Meta-Analyses extension for Scoping Reviews; CINAHL: Cumulative Index of Nursing and Allied Health; CENTRAL: Cochrane Central Register of Controlled Trials

Figure 1 presents the PRISMA-ScR flow diagram summarizing the study selection process. A total of 118 records were identified, and 18 records were assessed at the full-text level. Thirteen records met the inclusion criteria. After collating multiple reports arising from the same study cohort or experimental series, including the reports by Nissinen and Pitkänen [16,17], Wilson et al. [8,18], and Kwon et al. [11,19], 10 studies were included in the final review. Full-text exclusions were primarily due to a focus on chronic epilepsy unrelated to TBI, review articles without original data, or secondary analyses.

Characteristics of Included Evidence

**Figure 2:**
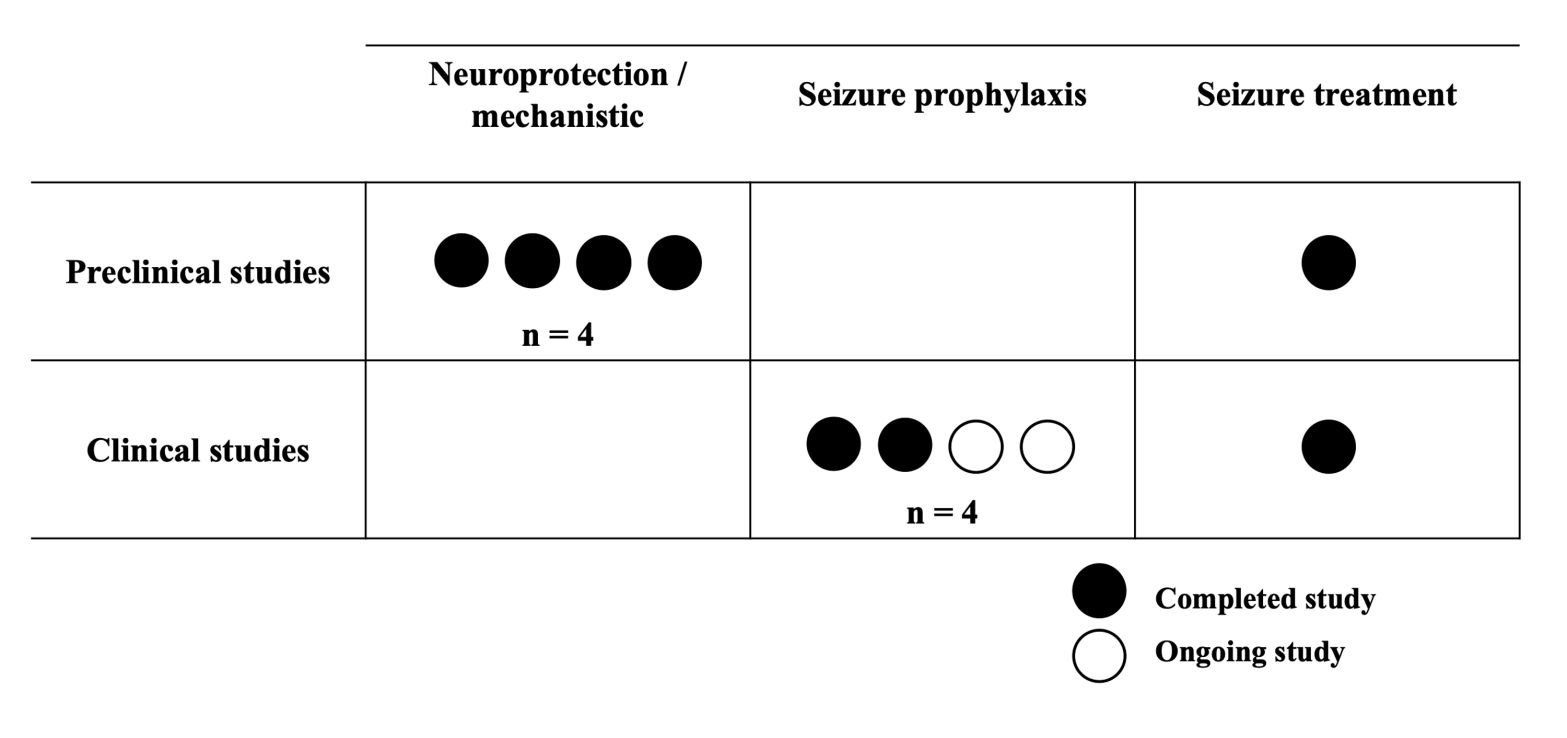
Evidence map of lacosamide use in traumatic brain injury-related seizure conditions. Studies shown in the map include preclinical studies by Nissinen and Pitkänen, 2006/2014 [16,17]; Wilson, 2012/2014 [8,18]; Wang, 2013 [9]; Mete, 2021 [10]; and Aykin, 2025 [20]. Completed clinical studies include Szaflarski, 2014 [21]; Kwon, 2017/2019 [11,19]; and Arai, 2024 [22]. Ongoing clinical studies include Ducote, 2024 [23] and Brintzenhoff, 2025 [13]. Studies are categorized by lacosamide indication (neuroprotection, seizure prophylaxis, or seizure treatment). Each symbol represents one study. Filled circles indicate completed studies, and open circles indicate ongoing studies. Empty areas indicate absence of identified studies.

Table 1 summarizes the characteristics of the 10 studies included in this scoping review. The body of evidence comprised both preclinical (n=5) and clinical (n=5) investigations, reflecting heterogeneous study designs and research contexts. Preclinical studies primarily employed experimental TBI models, including fluid-percussion, focal cortical injury, closed head injury, and weight-drop models. Clinical studies included two randomized controlled trials, two observational cohort studies, and a single case report conducted in acute care settings. The included clinical populations predominantly involved patients with moderate to severe TBI, often with intracranial hemorrhage or depressed consciousness at presentation. Figure 2 displays the distribution of preclinical and clinical studies according to LCM indication.

**Table 1:** Summary of preclinical and clinical evidence on lacosamide in traumatic brain injury. TBI, traumatic brain injury; RCT, randomized controlled trial; ASDH, acute subdural hematoma; CRMP-2, collapsin response mediator protein 2. *Indication: Neuroprotection includes studies evaluating lacosamide for injury-related preservation or mechanistic effects after traumatic brain injury, including neutral findings. Seizure prophylaxis refers to lacosamide use for prevention of post-traumatic seizures. Seizure treatment refers to lacosamide use for established seizures or status epilepticus after traumatic brain injury.

Author, year	Population/TBI model	Indication*	Key findings
Preclinical studies			
Nissinen & Pitkänen, 2006/2014 [16,17]	Rat/fluid-percussion injury	Neuroprotection	No neuroprotective effect on post-traumatic structural or cognitive outcomes; motor recovery was not improved and showed mild delay at later time points, with no overall compromise of structural integrity.
Wilson, 2012/2014 [8,18]	Rat/focal cortical injury models	Neuroprotection	Attenuation of post-traumatic axonal sprouting and excitatory synaptic connectivity via CRMP-2-related mechanisms; mechanistic evidence without demonstrated functional benefit.
Wang, 2013 [9]	Mouse/closed head injury	Neuroprotection	Improved functional recovery with reduced acute neuronal injury and neuroinflammatory responses.
Mete, 2021 [10]	Rat/weight-drop	Neuroprotection	Reduced oxidative stress and hypoxia-related injury, with partial normalization of electrophysiological activity.
Aykin, 2025 [20]	Rat/weight-drop with pentylenetetrazole	Seizure treatment	Reduced seizure severity in post-traumatic epilepsy, with enhanced seizure control when combined with adjunctive therapy.
Clinical studies			
Szaflarski, 2014 [21]	Severe TBI (RCT)	Seizure prophylaxis	Early neurological improvement (Glasgow Coma Scale trajectory) compared with phenytoin.
Kwon, 2017/2019 [11,19]	Severe TBI (Observational)	Seizure prophylaxis	Comparable early seizure prevention to phenytoin, with similar early post-traumatic seizure prevention rates and an improved safety profile, including fewer adverse drug events.
Arai, 2024 [22]	Elderly ASDH (Case)	Seizure treatment	Failure to control refractory nonconvulsive status epilepticus in elderly acute subdural hematoma.
Ducote, 2024 [23]	Adult TBI (Observational)	Seizure prophylaxis	Ongoing study: Observational evaluation of seizure prevention efficacy and safety vs levetiracetam.
Brintzenhoff, 2025 [13]	Moderate–severe TBI (RCT)	Seizure prophylaxis	Ongoing trial: Randomized evaluation of agitation and behavioral adverse effects vs levetiracetam.

How Lacosamide Was Used

Across the included studies, LCM use was categorized into three indications: seizure prophylaxis, seizure treatment (including status epilepticus), and neuroprotection or mechanistic investigation (Table 1). In preclinical studies, LCM was administered primarily to explore neuroprotective effects and injury-related pathophysiological mechanisms following experimental TBI, often as part of mechanistic or hypothesis-generating investigations. In clinical studies, LCM was used either for seizure prophylaxis in the early post-injury period or for treatment of established seizures. Characteristics of LCM administration varied across studies and were inconsistently reported, particularly with respect to how and when the drug was initiated and the use of comparator agents.

Outcomes Reported

In preclinical studies, reported outcomes primarily consisted of mechanistic and neurobiological measures, such as seizure frequency or latency, electroencephalographic parameters, and indices of neuronal network excitability. Additional outcomes included histological assessments reflecting neuronal injury or axonal changes, inflammatory markers, and behavioral measures used to evaluate injury-related and mechanistic effects. Seizure-related outcomes were reported less frequently in preclinical studies.

Among clinical studies, seizure-related outcomes, including early post traumatic seizures, post-traumatic epilepsy, and seizure control, were reported in all included studies. Safety and tolerability outcomes, such as adverse events and treatment discontinuation, were assessed in several investigations. Table 2 demonstrates variability in reported treatment characteristics, including timing of initiation, route of administration, maintenance regimens, and comparator protocols, whereas detailed loading strategies, titration schedules, and dose adjustments according to age or injury severity were often incompletely described. In contrast, neurological or functional outcomes, such as level of consciousness or functional recovery scales, were reported in only a subset of studies.

**Table 2:** Clinical studies evaluating lacosamide use in acute traumatic brain injury. TBI, traumatic brain injury; RCT, randomized controlled trial; IV, intravenous; GCS, Glasgow Coma Scale; PO, oral; PTS, post-traumatic seizure; ADE, adverse drug event; ASDH, acute subdural hematoma; NCSE, non-convulsive status epilepticus; NR, not reported. Notes: Lacosamide administration details are summarized at the study level; exact dosing regimens and dose adjustments were variably reported or incompletely described across studies.

Author, year	Population (TBI severity, n)	Study design	Lacosamide use	Comparator	Outcome and effect (as reported)
Szaflarski, 2014 [21]	Severe TBI (n=11)	RCT	IV; initiated in acute phase; duration seven days	Fosphenytoin	Improved GCS by Day 7 (p=0.05)
Kwon, 2017/2019 [11, 19]	Adult severe TBI with intracranial hemorrhage (GCS <9, n=481)	Observational	V or PO; initiated within 24 h; duration seven days; loading dose 200 mg in selected severe cases; maintenance 50–100 mg twice daily	Phenytoin	Early PTS: 1.4% vs 0.9% (p=1.00); ADEs: 0.5% vs 5.2% (p=0.003)
Arai, 2024 [22]	Elderly ASDH (n=1)	Case report	Route: NR; initiated on admission; maintenance 100 mg/day; duration NR	None	NCSE refractory to lacosamide
Ducote, 2024 [23]	Adult severe TBI (GCS <9, n NR)	Observational	Route: NR; initiated within 24 h; duration seven days; dose NR	Levetiracetam	NR (ongoing study)
Brintzenhoff, 2025 [13]	Moderate–severe TBI (estimated n≈600)	RCT	IV or PO; initiated within 24 h; duration seven days; maintenance 200 mg twice daily	Levetiracetam	NR (recruiting)

Evidence Gaps Highlighted by Mapping

Mapping of the included evidence demonstrated limited alignment between preclinical and clinical studies. Preclinical investigations predominantly evaluated neuroprotective and mechanistic outcomes, whereas clinical studies focused mainly on seizure-related and safety outcomes, resulting in limited overlap of outcome domains across experimental and clinical settings.

Within the clinical literature, heterogeneity was observed in TBI definitions, injury severity, and characteristics of LCM administration, including timing, route, dosage, and comparator treatments. Although seizure-related outcomes were consistently reported, neurological or functional outcomes were evaluated less frequently, and key methodological details were variably documented, limiting comparability across studies.

Discussion

This scoping review mapped preclinical and clinical literature examining the use of LCM after TBI. Studies were organized by study design, indication, and reported outcomes to describe the overall structure of the evidence base. Although evidence spans both experimental and clinical domains, alignment across indications, outcome domains, and timing of exposure remains limited.

Whereas preclinical studies have primarily focused on neuroprotective and mechanistic questions following experimental TBI, clinical studies have largely examined LCM use for seizure prophylaxis, seizure treatment, and safety or tolerability in acute care settings. In experimental models, LCM has been studied in relation to seizure-related physiology and neuronal network excitability, with additional assessments of injury associated changes; these include structural alterations [16,17], inflammatory responses [9], and electrophysiological measures [10,20]. Studies have also explored molecular and cellular mechanisms, such as CRMP-2-related pathways [8] and oxidative stress responses [10] within experimental TBI contexts.

In contrast, clinical studies have evaluated LCM primarily in pragmatic treatment contexts during the acute phase of TBI care. These investigations have focused on seizure occurrence, seizure control, and short-term safety, often in comparison with established antiepileptic agents such as phenytoin or levetiracetam [11,19,21]. More recent clinical reports and ongoing studies have continued to examine tolerability and feasibility in real world intensive care settings, including specific adverse effect profiles relevant to acute neurocritical care [13,22,23]. Overall, overlap between preclinical and clinical applications of LCM appears limited, reflecting differences in research purpose, outcome selection, and timing of exposure.

Clinical studies have generally examined LCM within the context of acute TBI management rather than as a primary efficacy comparator. Across studies, patient populations differed with respect to TBI definitions, injury severity, and inclusion criteria, resulting in variability in baseline seizure risk and neurological prognosis [11,21]. Patterns of LCM use also varied across investigations, including differences in timing of initiation, route of administration, dosing strategies, treatment duration, and choice of comparator agents. Several studies initiated LCM within 24 hours after injury, whereas others described treatment as beginning during the acute phase without precise temporal definitions. Comparator agents included phenytoin, fosphenytoin, and levetiracetam, situating LCM within established acute seizure management frameworks. Detailed treatment protocols were variably reported across studies.

The definition and timing of the acute phase after TBI were not uniform across the included studies. In clinical practice and guideline discussions, antiepileptic therapy is commonly framed around early post-traumatic seizures, typically defined as seizures occurring within the first seven days after injury [24]. However, operational definitions of this period varied across studies, resulting in differences in the temporal relationship between injury, seizure risk, and LCM exposure. This variability likely reflects differences in acute TBI care workflows and study design, in which treatment timing is influenced by stabilization priorities, neurological assessment, and intensive care logistics rather than intentional experimental contrasts [6].

Outcome selection differed systematically across the existing evidence on LCM use after TBI, reflecting differences in research objectives between experimental and clinical contexts. Clinical studies predominantly focused on outcomes relevant to acute neurocritical care, such as early post-traumatic seizures, seizure control, and safety or tolerability. As a result, the current evidence captures complementary but largely non-overlapping dimensions of LCM use after TBI.

This scoping review has several limitations. As a mapping review, the findings reflect the structure and reporting of the existing literature, rather than addressing comparative effectiveness. In addition, the number of available clinical studies and included participants was limited, which underscores the early stage of evidence development and highlights the need for future studies with clearer exposure definitions, aligned outcome domains, and adequately powered clinical designs.

Based on the structure of the existing evidence, future studies of LCM use in TBI may benefit from clearer alignment between study purpose, timing of exposure, and outcome selection. Greater consistency in how the acute phase is defined and reported could facilitate comparison across clinical studies and improve interpretability of findings. In addition, closer consideration of how experimental outcomes relate to clinically observable seizure and neurological outcomes may help bridge differences between experimental and clinical research contexts.

## Conclusions

The current evidence on LCM use after TBI is limited and heterogeneous. Preclinical studies emphasize mechanistic outcomes. Clinical studies focus on seizure-related and safety endpoints. Translational alignment remains limited. This review clarifies the evidence structure and identifies areas requiring coordinated investigation.

## Appendices

Supplementary appendix S1: search strategy

Database 1: MEDLINE (via PubMed)

Date of Search: December 2025

Search Strategy:

#1 (lacosamide OR vimpat)

#2 (brain injur* OR traumatic brain injur* OR TBI OR head trauma* OR head injur* OR brain trauma* OR cerebral trauma* OR craniocerebral trauma* OR brain damage*)

#3 #1 AND #2

Limits Applied: None

Database 2: CENTRAL (Cochrane Central Register of Controlled Trials)

Date of Search: December 2025

Search Strategy:

#1 (lacosamide OR vimpat)

#2 (brain injur* OR traumatic brain injur* OR TBI OR head trauma* OR head injur* OR brain trauma* OR cerebral trauma* OR craniocerebral trauma* OR brain damage*)

#3 #1 AND #2

Limits Applied: None

Database 3: CINAHL (via EBSCOhost) 

Date of Search: December 2025 

Search Strategy: 

#1 (lacosamide OR vimpat) 

#2 (brain injur* OR traumatic brain injur* OR TBI OR head trauma* OR head injur* OR brain trauma* OR cerebral trauma* OR craniocerebral trauma* OR brain damage*) 

#3 #1 AND #2 

Limits Applied: None

Database 4: ClinicalTrials.gov

Date of Search: December 2025

Search Terms Used:

lacosamide OR vimpat

(traumatic brain injury OR TBI OR head trauma OR brain injury OR brain damage)

Search Fields: All fields

Recruitment Status: All

Study Type: All

Database 5: Web of Science Core Collection

Date of Search: December 2025

Search Strategy:

TS = (lacosamide OR vimpat)

AND

TS = (brain injur* OR traumatic brain injur* OR TBI OR head trauma*

      OR head injur* OR brain trauma* OR cerebral trauma*

      OR craniocerebral trauma* OR brain damage*)

Supplementary appendix S2: data extraction instrument

The following fields were used for data extraction and were refined as needed to meet the review objectives. Extracted items included study characteristics (authors, year, country, design, publication type), population or model details (participant demographics, TBI type, sample size), and intervention parameters (LCM dose, route, timing, duration, comparators, and co-interventions). Outcomes included seizure prophylaxis, seizure treatment, or neuroprotection, as well as seizure incidence, neurological outcomes, adverse events, and molecular or histological findings.
